# Review: The Role of Dual-Energy Computed Tomography in Detecting Monosodium Urate Deposits in Vascular Tissues

**DOI:** 10.1007/s11926-024-01151-y

**Published:** 2024-05-13

**Authors:** Julia Held, David Haschka, Pietro G. Lacaita, Gudrun M. Feuchtner, Werner Klotz, Hannes Stofferin, Christina Duftner, Günter Weiss, Andrea S. Klauser

**Affiliations:** 1 Department of Internal Medicine II, Medical University Innsbruck, Innsbruck, Austria; 2 Department of Radiology, Medical University Innsbruck, Innsbruck, Austria; 3 Division of Clinical and Functional Anatomy, Department of Anatomy, Histology and Embryology, Medical University Innsbruck, Innsbruck, Austria

**Keywords:** Dual Energy Computed Tomography, Cardiovascular, Monosodium Urate, Gout, Hyperuricemia

## Abstract

**Purpose of Review:**

To highlight novel findings in the detection of monosodium urate deposits in vessels using dual energy computed tomography, and to discuss the potential clinical implications for gout and hyperuricemia patients.

**Recent Findings:**

Gout is an independent risk factor for cardiovascular disease. However, classical risk calculators do not take into account these hazards, and parameters to identify patients at risk are lacking. Monosodium urate measured by dual energy computed tomography is a well-established technology for the detection and quantification of monosodium urate deposits in peripheral joints and tendons. Recent findings also suggest its applicability to identify vascular urate deposits.

**Summary:**

Dual energy computed tomography is a promising tool for detection of cardiovascular monosodium urate deposits in gout patients, to better delineate individuals at increased risk for cardiovascular disease.

## Introduction

Gout is the most common inflammatory arthritis and its worldwide prevalence ranks between < 1%—6.8% and over the last years, the substantial burden of disease even increases without signs of levelling off [1–3]. In gout, persistent hyperuricemia exceeding the solubility threshold of uric acid in the circulation leads to monosodium urate (MSU) deposits in the articular and periarticular tissues, initially without clinical symptoms until the first gout flare occurs. If hyperuricemia persists, tophi may develop over time [4–6]. Acute gout flares take place when MSU deposits induce the innate immune response mainly via NLPR3 inflammasome activation resulting in painful arthritis or periarticular inflammation [7]. Despite the rising burden of the disease, disease management remains poor. This reflects the persistent premature mortality gap accompanied by gout diagnosis, which remained unchanged over the last two decades. This underscores the neglected role of gout as a driver of cardiovascular disease and its sequels [8]. Several studies address the driving effect of inflammation on arteriosclerosis. High sensitive C-reactive protein (CRP) or macrophage activation factor neopterin are a validated parameter to assess individual CV risk [9]. Of note, anti-inflammatory treatment such as the interleukin-1 beta blocker canakinumab or alkaloid colchicine have shown to reduce clinical endpoints of cardiovascular disease [10–12]. Although gout is associated with multiple known cardiovascular (CV) risk factors, such as reduced renal function, hyperlipidaemia or metabolic syndrome, there is no satisfactory explanation for the nearly doubled CV event rate observed in patients with gout [13]. Therefore, several studies suggest gout and even hyperuricemia might be independent risk factors for CV disease [14–22]. One hypothesis to explain this higher hazard in CV events is linked to the pro-inflammatory effects induced by intravascular MSU deposits. This theory has sparked controversies, as the presence of MSU in vessels is subject of debate, with some studies failing to prove the presence of MSU deposits [23], while others have detected classical needle shaped, negatively birefringent crystals as evidence for uric acid accumulation inside arteriosclerotic plaques in coronary arteries [24••, 25, 26]. Detection of these crystals via microscopic examination is the gold standard in peripheral joints and tophi to confirm MSU deposition. However, examination in solid tissues brings some queries. Formalin fixation leads to dissolution of the crystals, ethanol fixation or staining also reduces crystal concentration, which can lead to false negative results despite being considered as a gold standard [27]. To overcome these problems and to avoid invasive diagnostic procedures, dual energy CT (DECT), an imaging technology that performs simultaneous acquisition at two energy levels to discriminate via specific radiographic attenuation uric acid, has proven to be a more sensitive than X-ray and Ultrasound in the detection of MSU in peripheral joints [28].

The importance of DECT is reflected by the 2015 EULAR/ACR classification criteria for gout, were gout diagnosis is enabled without invasive joint aspiration using clinical and laboratory parameters as well as DECT beside ultrasound and x-ray [29]. As a well-established tool to detect peripheral MSU deposits, its application on the vascular system has recently been proven feasible [30–33].

Herein, we discuss the current knowledge and available literature of CV MSU deposits investigated by DECT and highlight future implications and possible limitations of this imaging technology.

## Search Strategy and Selection Criteria

A search in PubMed up to March 15th, 2024 queried the terms “dual energy computed tomography/DECT and cardiovascular/vasculature” and “Urate/Monosodium urate and vascular/cardiovascular imaging”. (Supplemental Text 1).

**Fig. 1:**
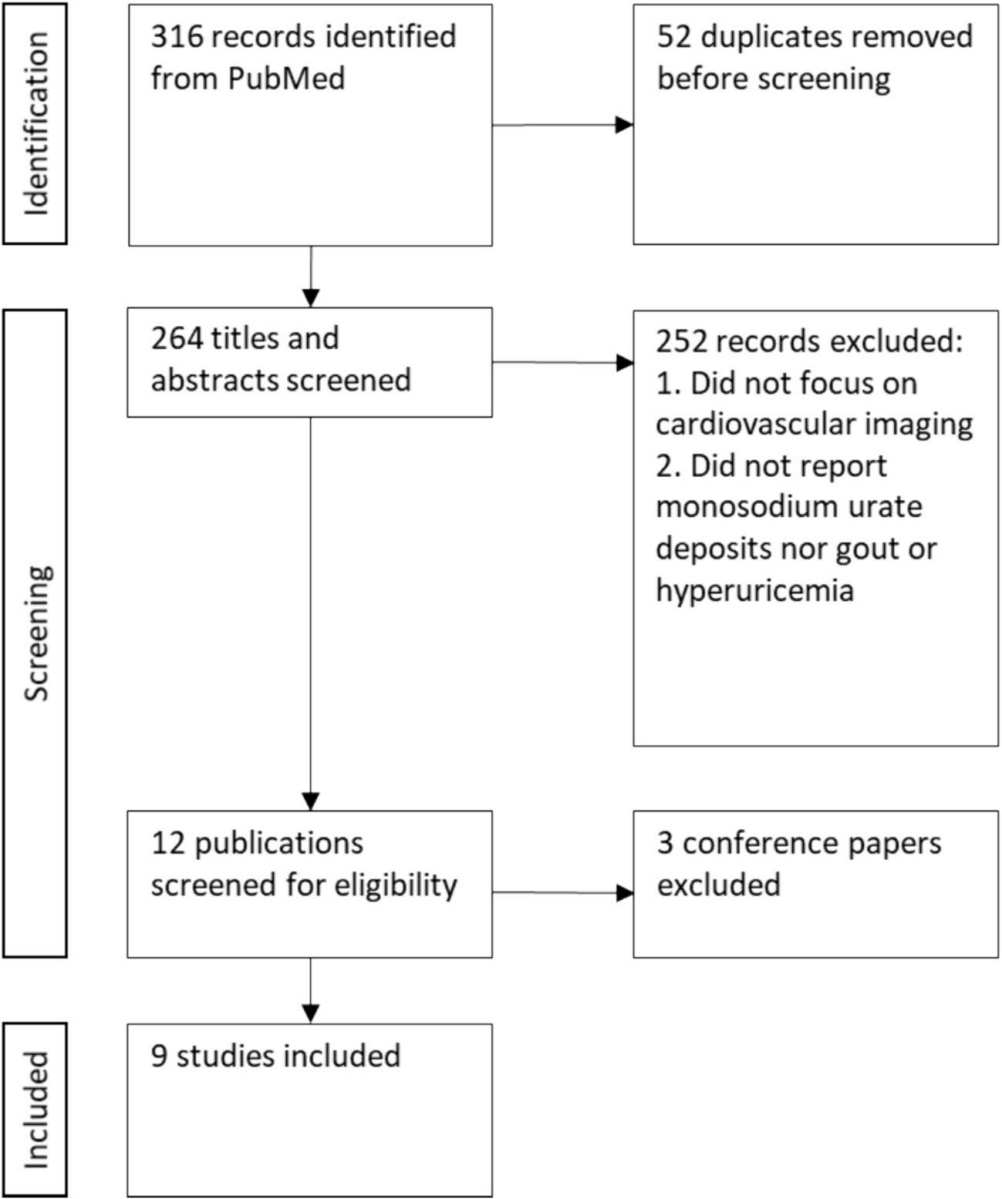
Identification, screening and inclusion criteria

This screening strategy identified 316 records, from which 264 titles and abstracts remained after removing duplicates for further examination. After excluding abstracts that did not pertain to randomized controlled trials, meta-analyses, cohort studies, cross-sectional, or case–control studies and upon elimination of abstracts not focusing on cardiovascular or vascular MSU deposits, 12 titles remained. Three publications were categorized as conference papers and were thus disregarded, resulting in 9 articles remaining available for this review (Fig. 1) (Table 1).

**Table 1:** Dual energy computed tomography in the detection of monosodium urate deposits

	Population	N	Study type	Outcome measures	DECT Findings
Kim et al. (2018)	Metabolic Syndrome and hyperuricemia	44	cross-sectional	Association between SUA levels and coronary flow reserve and urate deposits in carotid arteries	No MSU deposits in carotid arteries. No significant association between SUA and CFR (β = − 0.12, p = 0.78) or stress MBF (β = − 0.52, p = 0.28)
Klauser et al. (2019)	Gout, controls and cadavers with unknown clinical history	112	prospective	Detection of peripheral and CV MSU deposits in aorta, coronary arteries, mitral and tricuspid valve by DECT, crystal characterisation with compensated polarizing light microscopy	CV MSU deposits were significantly higher in gout patients. (86.4% vs. 14.9%, p < 0.001). In three cadavers polarized light microscope verified MSU deposits
Barazani et al. 2020	Gout and controls	49	prospective	Detection of CV MSU deposits in aorta and coronary arteries by DECT	Gout patients had significantly higher MSU volumes in aorta, 55% of gout patients had coronary MSU deposits compared to 0% of controls
Pascart et al. 2021	Gout and controls	152	prospective	Detection of knee and popliteal artery MSU deposits by DECT	No difference between gout and controls. MSU positive plaques in the lower extremities in 24.6% vs. 23.1% (n.s.), no effects of ULT
Feuchtner et al. 2021	Gout, hyperuricemia and controls	96	prospective	Detection of peripheral and CV MSU deposits in aorta, coronary arteries, mitral and tricuspid valve by DECT	DECT positive MSU plaques in over 90% of gout patients and one third of the hyperuricemia patients. Only one DECT positive MSU in controls
Dalbeth et al. 2022	Explanted Aorta of cadavers, unknown clinical history	6	prospective	Detection of aortic CV MSU deposits by DECT and compensated polarizing light microscopy	No MSU crystals detected neither in DECT nor in microscopy
Klauser et al. 2022	Cadavers, embalmed and fresh, unknown clinical history	49	prospective	Detection of peripheral and CV MSU in cranium, neck, body trunk and feet by DECT and compensated polarizing light microscopy	80.5% of cadavers showed MSU positive plaques in aorta, no MSU positive plaques were detected in coronaries or intracerebral vessels. MSU deposits confirmed by microscopy in 10/10 biopsies
Ren et al.2024	Angina pectoris symptomatic with and without gout	872	prospective	Detection of coronary CV MSU deposits by DECT	Presence of CV MSU deposits were higher in patients with calcified plaques than in those without (19% vs. 1.9%). Patients with MSU positive CV plaques found more often in gout patients (25.7%vs 10.4%)
Yokose et al. 2024	Gout and controls	106	retrospective	Detection of CV MSU deposits in aorta superior, vena cava, right atrium and coronary arteries by DECT	Retrospective postprocessing of pulmonary embolism scans showed MSU coded plaques in 85% of gout patients and 84% of controls. Advanced DECT measurements using Rho/Z_eff_ detected all green spots as artefacts. No ECG triggering known to cause artifacts

Overview of included studies in this review. DECT = Dual energy Computed Tomography, SUA = Serum urate Acid, MSU = monosodium urate, CFR = coronary flow reserve, MBF = myocardial blood flow, CV = cardiovascular, ULT = urate lowering treatment

## Detection of CV MSU depositions

The very first attempt to detect vascular MSU depositions with DECT was in 2018. Kim et al. investigated 44 patients with metabolic syndrome und hyperuricemia (serum uric acid levels (SUA) > 6.5mg/dl) to assess a possible association between SUA-levels, coronary flow reserve and carotid artery MSU deposits. Gout patients were excluded in this study. This study cohort showed no association of SUA with coronary flow reserve, and none of the investigated carotid arteries revealed DECT positive MSU coded plaques [34].

**Fig. 2:**
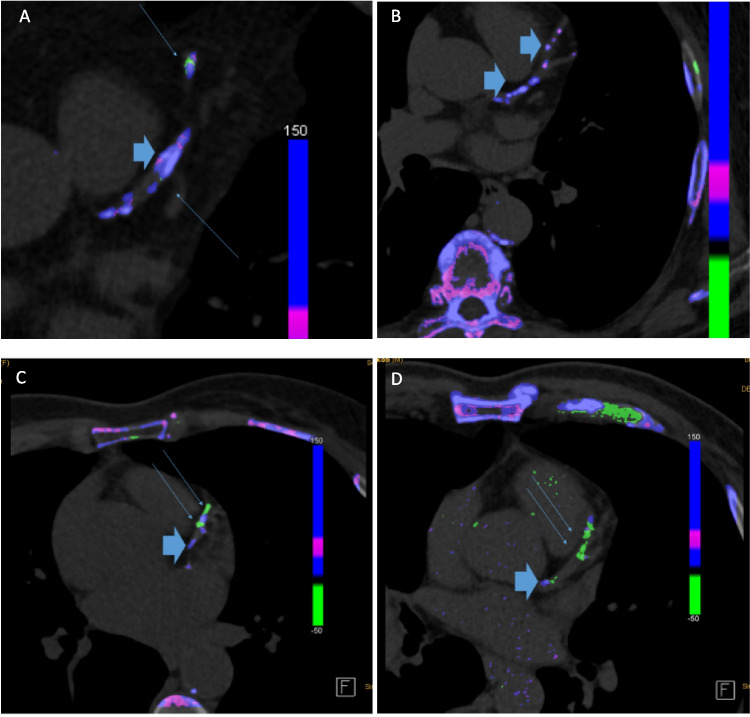
A: Calcified plaque (thick arrow) without MSU deposits, green spots (small arrow) are classified as artefacts. B: Calcified plaque without any MSU deposits (thick arrow) C: Mixed plaque with green (MSU) and calcific deposits D: Mainly MSU associated plaque only minor calcifications

Three other studies investigated gout patients and controls with DECT. All three detected MSU positive plaques by DECT in the CV system. Barazani et al. found a significant higher volume of MSU within the aorta in 31 gout patients compared to controls, whereas no difference in the number or total volume of MSU coded plaques in DECT was observed between patients with either tophaceous or non-tophaceous gout. In coronary arteries, MSU coded plaques were found in 54% (n = 15/29) of gout patients compared to no MSU deposits in the control group (p < 0.001) [35]. Feuchtner et al. investigated 96 patients, 37 gout, 33 hyperuricemia and 26 controls. 91.9% of gout patients showed MSU positive plaques compared to 3.8% of the control group (p < 0.0001). Of 102 MSU positive plaques, one third (26.7%) were only MSU positive, the other 2/3 (74.2%) were mixed MSU positive and calcified, and MSU positive plaques were more prevalent in gout compared to hyperuricemia and control patients (91.6% vs 2.9% vs 3.8%, p < 0.001 respectively). (Figs. 2: Examples of calcified plaques, mixed plaques and only MSU plaques) Furthermore, in this cohort, coronary calcium score was higher in patients with gout compared to controls (659.1 vs 112.4 Agatston score; p < 0.001). Additionally, the authors performed an ex vivo phantom study. They scanned MSU crystal solutions of 5%, 10%, 15%, 20% and 25%. MSU deposits could be verified by DECT at concentrations of 15% or higher. Moreover, a model with hydroxylapatite as a calcified plaque model was clearly different from the MSU model upon DECT analysis [36•].

Most recent, Ren et al. investigated 872 patients with angina pectoris symptoms. 50.6% (n = 441) of patients had coronary plaques, 348 (78.9%) out of them had atherosclerotic plaque, 8 had MSU positive depositions, and 85 mixed MSU positive and atherosclerotic plaques. In the multivariable analysis, MSU deposition was independently associated with plaques after adjustment for age, sex, blood pressure, blood glucose, serum creatinine, history of gout, and history of hyperuricemia (OR = 13.69, 95%CI: 7.53–22.95, P = 0.035) [37•].

Pascart et al. investigated popliteal arteries in 126 gout patients and 26 controls by DECT. In this cohort, prevalence of DECT based MSU positive plaques were equally distributed between gout and control group. As previously reported, presence of MSU coded plaques and arterial calcification were significantly associated (p < 0.001). In contrast to other studies, the authors concluded that the association with calcifications might affect the reliability of DECT measurements by allowing for artefacts and that MSU coded plaques might not reflect true MSU deposits [38•]. How these findings in the popliteal artery might serve as parameters to assess individual CV risk should be further investigated.

Focusing on artefacts in DECT measurements, Yokose et al. reported a retrospective analysis of pulmonary DECT scans in 106 patients. The scans were postprocessed using the raw data from scans performed due to the clinical suspicion of pulmonary embolism. The scans were non-ECG-gated. Of 106 patients 48 were classified as gout patients: 10 had confirmed gout, 7 probable gout as diagnosed by an rheumatologist, 31 had either uric acid lowering treatment or chart review based diagnosis or elevated serum uric acid (SUA) above 6mg/dl. Green spots indicating MSU in the vasculature were observed in 85.2% of the gout patients scans and in 84.3% of controls. The aim of the group was to differentiate between artefacts and true MSU deposits by advanced DECT measurement, using electron density (Rho) and effective atomic number (Z_eff_). This approach defined all green spots as streak, contrast medium mixing, foreign body, noise or motion artefacts, more often found in non -ECG -gated CT [39•].

In order to verify MSU-deposits detected by DECT, compensated polarized light microscopy (CPLM) was performed by a study of Klauser et al. investigating gout, hyperuricemia and control patients. All 106 patients underwent thoracic CT Scans. DECT and coronary calcium score were performed and revealed DECT positive MSU deposits in 86.4% (n = 51) of gout patients compared to 14.9% (n = 7) in the control group. Respectively, the coronary calcium score was significantly higher in gout patients as compared to controls (950 AU; 95% CI, 639–1261 vs. 217 AU; 95% CI, 37–397, p < 0.001). Interestingly, there was no association between SUA and CV MSU deposits in the gout cohort. To verify the DECT-findings, six fresh cadavers with unknown clinical history were examined. Three out of six analyses revealed DECT positive MSU deposits and were further investigated. MSU positive plaques visualized by DECT were histological proven by polarized light microscopy. 7 of 8 biopsies showed crystals with the typical needle like appearance and strong negative birefringence, typical characteristics of MSU (positive predictive value of DECT = 87.5%) [24••].

**Fig. 3:**
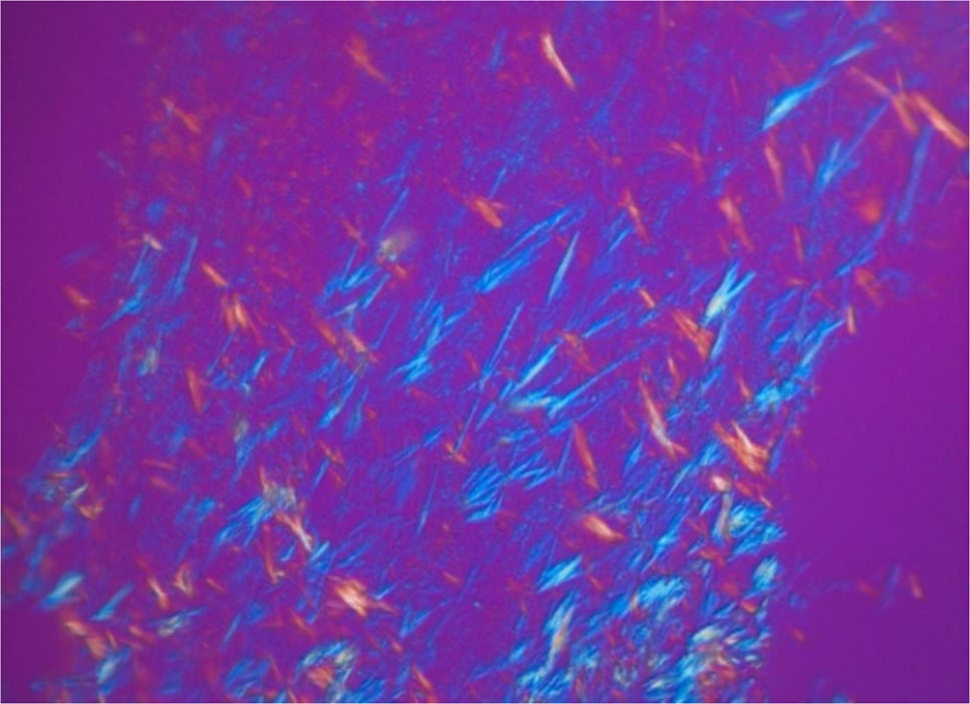
Compensated polarized light microscopy of aortic plaques showing the classical bifringent properties of uric acid crystals

These finding were further confirmed in a cadaver study from Klauser et al. in 2022. 41 embalmed cadavers and 8 fresh cadavers were investigated. 33 of the 41 (80.5%) embalmed cadavers showed MSU-positive vascular deposits within the aorta. Of the fresh cadavers, one showed MSU deposits in the thoracic aorta. CPLM confirmed vascular MSU deposits in 10/10 biopsies [40•] (Fig. 3).

A second cadaver study from Dalbeth et al. showed negative results in 6 aortas, no DECT positive plaques were detected. They performed CPLM of the DECT negative plaques confirming the absence of MSU typical crystals [41••].

## Discussion

Increased CV mortality remains an unsolved problem in patients with gout and hyperuricemia [8]. Therefore, better stratification tools are needed to identify patients at risk and to close the mortality gap. CV MSU deposits may provide valuable information to identify patients at risk for major adverse cardiac events [42].

**Fig. 4:**
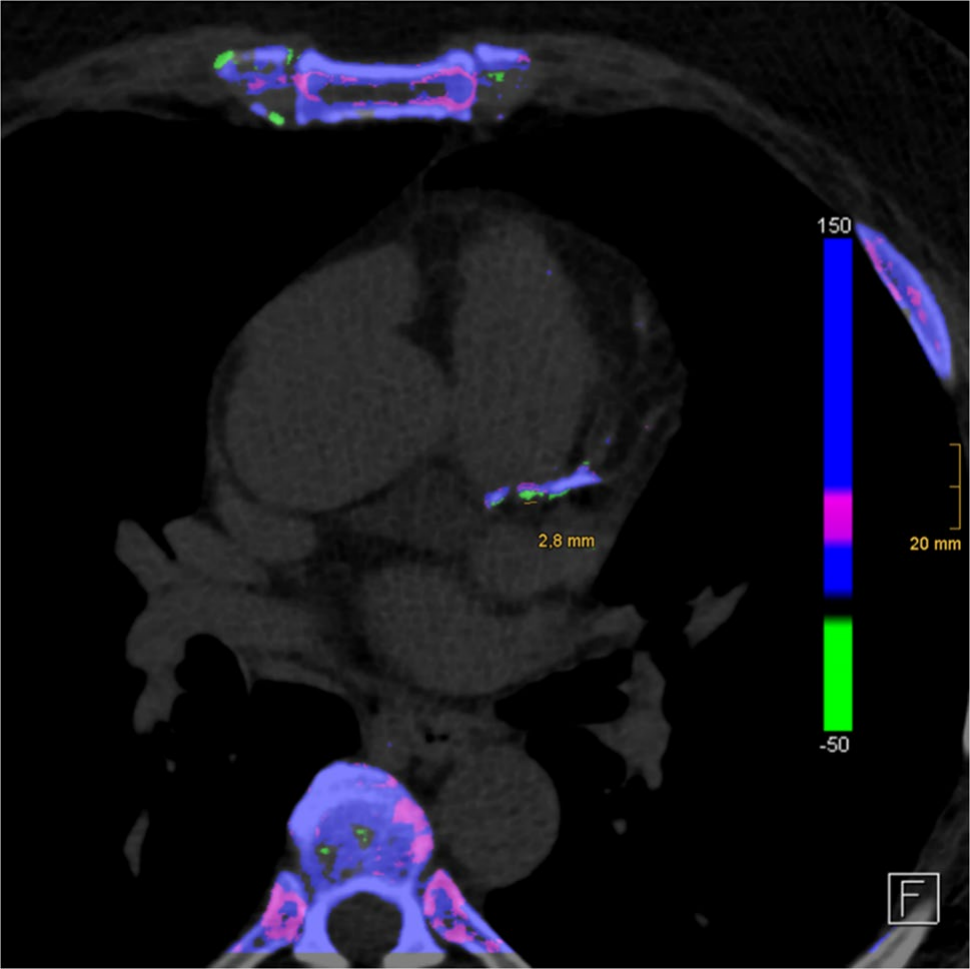
Green spots smaller than 3 mm rated as artefacts

Direct crystal evaluation in the CV system is often not feasible. DECT offers a useful tool to detect these MSU deposits. DECT demonstrates high accuracy in distinguishing crystal deposits, as MSU crystal DECT ratios are significantly different from other crystals like calcium pyrophosphate and calcium hydroxyapatite [43]. However, artefacts can occur in every imaging modality. Artefacts have to be excluded in the interpretation, which experienced investigators achieve by using a threshold of 3 mm and adequate protocols (Fig. 4).

Especially in cardiac imaging, ECG-gated cardiac protocols should be used to ensure highest spatial resolution with minimal motion artefacts. The enhanced resolution and improved dose efficacy of new CT scans, such as photon counting detector CTs (PCD-CT), necessitate reconsideration of the current 3 mm cut-off for MSU deposit detection [44, 45]. PCD-CT is a novel technology that was commercially introduced for the first time in 2021. PCD-CT is based on a different detector technology, directly converting X-rays into an electrical signal [46], thereby minimizing electronic noise. Furthermore, a higher spatial resolution of 0.16–0.2mm^2^ is obtained. PCD-CT has shown to improve image quality by reducing artifacts and enhancing contrast-to-noise ratio, while reducing radiation and contrast agent volume by up to 50% [47]. Most importantly, ultra-high-resolution (UHR) imaging is feasible with PCD-CT, allowing for improved visualization of smaller structures such as coronary plaque and a reduction of the “calcium blooming” artifact. Initial in-vitro and in-vivo studies have demonstrated improved accuracy of PCD-CT for stenosis grading compared to conventional energy-integrating detector CT (EID-CT) [48]. Additionally, PCD-CT provides a special image reconstruction algorithm (Purelumen™) using spectral imaging for automated virtual calcium removal from the coronary vessel wall. This algorithm has shown promising initial results in 30 patients, with higher accuracy for stenosis grading compared to conventional evaluation [49]. All these technological advances offer promising potential for PCD-CT in more accurately characterizing coronary plaques, including lesions containing MSU crystals.

**Fig. 5:**
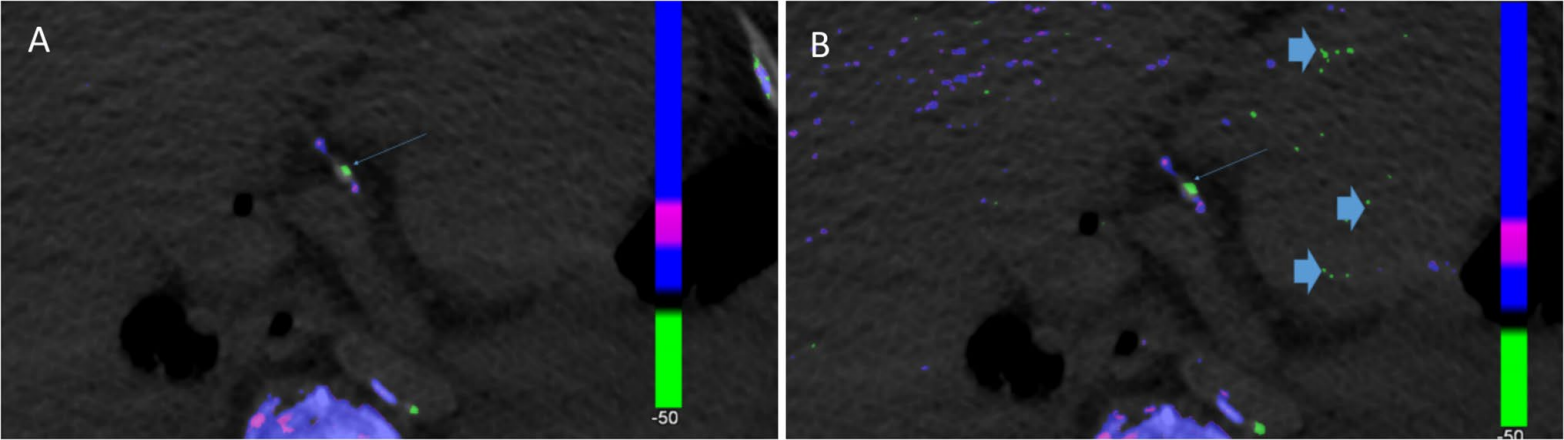
Postprocessing showing better small MSU deposit in a plaque compared to standard processing. Note: However also artefacts (thick arrows) are increasing A: Standard postprocessing, B. Postprocessing according Strobl et al. [51]

In two studies investigating peripheral MSU deposits more sensitive postprocessing protocols than standard DECT settings were used in order to assess presence, size and amount of MSU deposits. It has been found that false negative findings of DECT could be overcome, with increased sensitivity and specificity when compared to high-resolution ultrasound and verification with polarising light microscopy. Naturally, artefacts also increase with the use of more sensitive post-processing methods, but these can be well distinguished by experienced examiners. This opens up further discussion whether post-processing techniques could also increase the detection rate of MSU deposits in the vasculature, which should be proven in further studies [50, 51] (Fig. 5).

Additionally, there is a lack of information about comparability and reproducibility of results across different CT machines.

When screening for CV MSU, patient’s selection appears crucial. Prevalence of CV MSU differs exceptionally in different cohorts. In confirmed gout according to the 2015 ACR/EULAR classification criteria, presence of vascular MSU varies between 25 and 90%. These percentage declines dramatically down to 0% when unbiased screening other cohorts like patients with metabolic syndrome, suspected pulmonary embolism or unstable angina pectoris without gout or hyperuricemia is performed [34, 41••]. Furthermore, the vessels investigated appear to impact the detection of CV MSU deposits. Peripheral vessels, such as the popliteal artery investigated in the VASCURATE study [38•], where a threshold of ≤ 2 mm for green spots was used, did not show differences between gout and control patients, suggesting that the observed high number of artefacts might be reasonable. The potential association of MSU deposits on popliteal arteries with MSU deposits in coronary arteries and its association with CV disease needs to be further elucidated. Advanced DECT measurements like Rho/Z_eff_ as reported by Yokose et al. might be a good tool to distinguish between artefacts and true MSU deposits [39•]. These findings must be further investigated, especially in larger longitudinal gout cohorts with ECG-gated imaging protocols and applicability of Rho and Z_eff_ in mixed plaques with not only MSU deposits are unknown.

So far, the gold standard in gout diagnosis is compensated polarized light microscopy (CPLM). MSU crystals are characterized by a needle-like shape and strong negative birefringence. CPLM converts this optical property of MSU crystals into colour variations. This method is error-prone, as low crystal concentrations lead to false negative results and the method is highly investigator dependent. In solid tissue like CV plaques, there are even more obstacles to overcome. If specimen can be obtained, fixation before microscopic examination is necessary. Formalin fixation is known to dissolve MSU crystals, and even ethanol fixation before staining reduces crystal concentration. Currently, the best method for specimen preservation is the use of frozen sections [27]. All these queries in crystal detection highlight the need for alternative methods. One potential future tool is Raman spectroscopy (RS). RS is a method in which light can either be absorbed or scattered when a photon stimulates a molecule in the investigated material. A fraction of the scattered light undergoes an energy shift compared to the source beam. By plotting the scattered light against frequency, a Raman spectrum is obtained, which serves as a unique identifier of the molecular structure of the material [52]. The combination of RS and polarized light showed increased objectivity compared to polarized light microscope alone, with persistent high specificity and sensitivity [53]. Initial attempts of in vivo MSU detection with RS showed promising results, with sensitivity comparable to ultrasound scans, although only the ventral aspect of the joint was investigated [54]. These attempts might indicate potential future implications for superficial vessels, like carotid arteries.

## Conclusion

The growing attention towards CV DECT will contribute to further evidence, but central questions remain unanswered:

- Is the number, volume or localisation of MSU deposits associated with a higher CV risk?
- Can stringent SUA-control reduce CV events in patients with CV MSU deposits detected by DECT?
- Is whole body DECT imaging, despite the limitation of increased radiation dose, beneficial for gout and hyperuricemia patients to measure the burden of gout and changes MSU load under therapy measured by DECT volumetry?
- Will new technologies (photon counting CT, Rho/Z_eff,_ RS) improve the detection of patients at risk?

This review points out the urgent need for further research to expand our understanding and enhance treatment and preventive healthcare options for gout patients.

## Data Availability

No datasets were generated or analysed during the current study.
